# Bmal1 Regulates Macrophage Polarize Through Glycolytic Pathway in Alcoholic Liver Disease

**DOI:** 10.3389/fphar.2021.640521

**Published:** 2021-03-10

**Authors:** Yiwen Zhou, Meifei Wu, Lei Xu, Jieling Cheng, Jie Shen, Tianyu Yang, Lei Zhang

**Affiliations:** ^1^ School of Pharmacy, Anhui Medical University, Hefei, China; ^2^ Inflammation and Immune Mediated Diseases Laboratory of Anhui Province, Hefei, China; ^3^ The Key Laboratory of Anti-inflammatory and Immune Medicines, Ministry of Education, Hefei, China

**Keywords:** Bmal1, S100A9, glycolysis, macrophage polarize, alcoholic liver disease

## Abstract

Hepatic macrophages play a critical role in inflammation caused by alcohol feeding. During this process, variation of macrophage phenotypes triggers inflammatory responses in a variety of ways. Moreover, there is increasing evidence that Brain and Muscle Arnt-Like Protein-1 (Bmal1) is regarded as a key regulator of macrophage transformation. In our study, Bmal1 was detected to be low expressed in EtOH-fed mice tissue samples and ethanol-induced RAW264.7 cells. After hepatic specific overexpression of Bmal1, M1 macrophage markers were evidently down-regulated, while M2 markers were on the contrary, showing an upward trend. Furthermore, alcoholic liver lesions were also improved in alcohol feeding mice with overexpressed Bmal1. On this basis, we also found that the glycolytic pathway can regulate macrophage polarization. *In vitro*, blocking of glycolytic pathway can significantly inhibit M1-type polarization. Importantly, glycolysis levels were also restrained after Bmal1 overexpression. What’s more, Bmal1 exerts a negative regulatory effect on glycolysis by interacting with S100A9 protein. Further studies showed that the alleviation of alcoholic liver disease (ALD) by Bmal1 was associated with glycolytic pathway suppression and M1 macrophage polarization. In summary, we demonstrated that Bmal1 is a gene capable of relieving ALD, and this effect may provide new insights for altering macrophage phenotypes to regulate inflammatory responses in ALD.

## Introduction

At present, liver disease has developed into the leading cause of diseases and death worldwide. Among them, ALD developed rapidly (Wang et al., 2014). Long-term excessive drinking leads to structural abnormalities and dysfunction of hepatocyte, which further progresses to ALD. The initial manifestation of the disease is alcoholic fatty liver disease (AFL) with hepatocyte steatosis, necrosis, regeneration and so on a series of changes (Liu, 2014). It is well-known that inflammation, oxidative stress, drinking patterns, viral infection and cell damage are typical drivers of alcoholic liver injury. More importantly, persistence of chronic inflammation, contribute to the progression of alcoholic fibrosis and cirrhosis (Wilfred De Alwis and Day, 2007; Louvet and Mathurin, 2015). Alcohol increases intestinal permeability, with Kupffer cells producing large amounts of the pro-inflammatory cytokine TNF-α, IL-1β, leading to the aggravation of ALD (Tsukamoto and Lu, 2001; Miller et al., 2011; Tilg et al., 2011; Lívero and Acco, 2016). Clinical research also demonstrates that TNF-α antagonists can improve liver function of patients with alcoholic hepatitis (Lopetuso et al., 2018). Although the above evidence suggests that inflammation may play a role in the pathogenesis of ALD, the specific regulatory mechanism remains unclear.

After a long period of research, macrophages make a difference to the initiation, maintenance, and resolution of inflammatory responses (Essandoh et al., 2016; Cochain and Zernecke, 2017; Murray, 2018). There is a compelling evidence that liver-specific macrophage activation is the core component in the course of ALD. Moreover, M1/M2 macrophage balance polarization governs the fate of an organ in inflammation or injury (Ti et al., 2015; Shapouri-Moghaddam et al., 2018). Studies have shown that there may be complex patterns of macrophage shape and function in the pathogenesis of inflammatory diseases such as non-alcoholic fatty liver disease (Alisi et al., 2017). LPS and IFN-γ promote classical M1-type macrophage differentiation and produce high levels of proinflammatory macrophage factors. IL-4 and IL-13 induced M2 macrophages have highly effective phagocytic activity and increase the production of anti-inflammatory cytokines. Alcohol as one of the obvious external stimulating factors, the aggregation of liver M1/M2 macrophages occurred after stimulation (Saha et al., 2015). It is worth noting that the metabolic state of immune cells is closely related to phenotypic changes, among which macrophages are the most obvious (Galván-Peña and O’Neill, 2014). M1 macrophages induced by LPS are characterized only by accelerated glycolysis. However, in M2 macrophages stimulated by IL-4, the oxidative phosphorylation rate was boosted (Wang et al., 2019). Hence, we consider that the glycolytic pathway affects M1 polarization of macrophages and participate in the regulation of inflammatory response.

As an internal rhythm regulating biological activities, the biological clock can trigger the body to make adaptive adjustment to the alteration of the external environment. However, Bmal1, which plays a central orchestration role in the molecular clock, is certainly involved in the process (Early et al., 2018). Based on previous studies, impaired liver Bmal1 expression or function may lead to chronic hepatic metabolic disease like ALD (Zhang et al., 2018). Crucially, researches have displayed that circadian rhythm and metabolic cycle in a tight coupling state, maintaining glucose metabolism homeostasis in mammals (Peek et al., 2013). Elevation of Bmal1 in human astrocytes has been reported to result in decreased expression of HK1 and LDHA proteins to inhibit aerobic glycolysis and lactic acid release (Yoo et al., 2020). Other studies also noted that the expressions of lactic acid and glycolysis augmented rapidly in the Bmal1^−/−^ liver (Peek et al., 2017). Therefore, Bmal1 protein is involved in a variety of physiological processes, especially energy metabolism processes such as glycolysis. However, the mechanisms by which Bmal1 regulates aerobic glycolysis still unclear in ALD.

In this article, we describe a pivotal role for Bmal1 in macrophage energetic regulation. After inflammatory stimulation, Bmal1 expression was blocked in macrophages. Its functional deficiency can promote glycolytic pathway and further accelerate the occurrence of M1 macrophage polarization. We observed a downregulation of Bmal1 levels in EtOH-fed mice, so we attempted to determine the potential role of Bmal1 in macrophage polarization and the molecular mechanism of this regulatory effect in ALD.

## Materials and Methods

### Mouse Model of Alcoholic Liver Disease

All experimental procedures were approved by the Animal Ethics and Use Committee of Anhui Medical University. Male C57BL/6J mice aged 6–8 weeks were obtained from the Experimental Animal Center of Anhui Medical University for ALD modeling. Mice were randomly divided into normal group and experimental group. We use the National Institute on Alcohol Abuse and Alcoholism (NIAAA) recommended method of Lieber-DeCarli (LD) liquid diet and alcohol intragastric administration to construct ALD mice model. We purchased animal feed from TROPHIC Animal Feed High-Tech Co. Ltd. (Hai’an, Jiangsu, China). The modeling process lasted a total of 16 days, including the liquid diet adaptation stage (5 days), modeling (10 days), gavage (1 time), and specimen (1 day). The EtOH-fed mice were randomly fed LD liquid diet containing 5% ethanol (5% v/v) for 10 days, and then ethanol was given separately according to their weight by gavage, while the control mice were ingested maltodextrin with the same amount of gavage. The freshly prepared mouse feed was changed every day as required. The mice were anesthetized 9 h later after the last intragastric intake of ethanol, and blood and liver tissues were collected for subsequent analysis. The extracted plasma was stored at −80°C. Some liver tissues were frozen immediately, while others were fixed in 10% formalin for subsequent experiments such as H&E and Oil red O staining. The current study was evaluated and approved by the Animal Care and Use Committee (number: LLSC20200977).

### Serum Levels of TG, TCH Assay and ALT/AST Activity Analysis

Alanine aminotransferases (ALT, C009-1–-1) assay kit, aspartate aminotransferases (AST, C010-1-1) assay kit, triglyceride (TG, A110-1-1) and total cholesterol (TCH, A111-1-1) assay kits were all from Jiancheng Institution PeproTech (Nanjing, Jiangsu, China). According to the instructions, TG and TCH levels were detected by TG and TCH assay kits in serum of EtOH-fed mice. Serum levels of ALT and AST in ALD mice were verified by ALT and AST activity detection kits, as recommended by the manufacturer.

### Immunohistochemistry

Liver tissue of each mouse which was fixed in 10% neutral buffered formalin solution was paraffin-embedded before routine histological staining. The prepared slides were dewaxed with xylene, dehydrated with alcohol, and then microwaved with sodium citrate buffer for 15 min to obtain the antigen. 3% hydrogen peroxide was incubated for 10 min to eliminate endogenous peroxidase activity after antigen repaired. These sections were then incubated overnight with a primary antibody against Bmal1 (1:200, ab272705, Abcam, United Kingdom) at 4°C. After washing with PBS, the sections were incubated with HRP-labeled broad-spectrum secondary antibody at room temperature for 20–30 min. The expression of Bmal1 was observed by 3,3′-diaminobenzidine tetrahydrochloride (DAB) staining. The sections were dehydrated after re-stained with hematoxylin for 3 min and then observed the positive region distribution of Bmal1 in the site of alcoholic liver injury.

### Immunofluorescence Staining

Sections were treated with 10% bovine serum albumin (BSA) blocking solution to avert nonspecific staining. We incubated the sections with Bmal1 rabbit polyclonal primary antibody and CD68 mouse monoclonal primary antibody at 4°C overnight. Then, the anti-rabbit FITC mixture and anti-mouse Cy-3- conjugated secondary antibody were added and incubated at room temperature for 2 h. Finally, adding 3,3′-diaminobenzidine tetrahydrochloride (DAB) staining to display the expression of Bmal1 and CD68. The stained sections were observed under an inverted fluorescence microscope (OLYMPUS IX83, Tokyo, Japan).

### Cell Culture

RAW264.7 cell line was acquired from the Type Culture Collection of the Chinese Academy of Sciences (Shanghai, China). The cells were grown in Dulbecco’s modified Eagle’s medium (DMEM, Hyclone, United States) added with 10% fetal bovine serum (FBS, Biological Industries, Israel), and cultured at 37°C containing 5% CO_2_ in an incubator. RAW264.7 cells grown in DMEM medium were stimulated by lipopolysaccharide (LPS, 1 µg/ml, L2880, Sigma-Aldrich, St. Louis, MO, United States) for 24 h to polarize M1 macrophages, while M2 macrophages were induced by IL-4 (15 ng/ml, CK74, novoprotein, shanghai, China) treatment for 24 h.

### Glycolytic Analysis

The extracellular acidification rate (ECAR) was monitored in real time by XF-24 Extracellular Flux Analyzer (Seahorse Bioscience). Cells with a density of 40,000 cells/well were inoculated into specific seahorse XF-24 cell culture microplates. According to the manufacturer’s operating manual, 10 mM glucose, 1 mM oligomycin, and 50 mM 2-deoxyd-D-glucose were added successively, recording the corresponding ECAR value of each point.

### Co-IP Assays

LPS induced-RAW264.7 cells were lyzed according to the instructions of the Co-Immunoprecipitation Kit (Co-IP Kit, BersinBio, Guangzhou, China). The precipitated Bmal1 and S100A9 immune complexes were obtained by coupling protein A/G-MagBeads with anti-Bmal1 and anti-S100A9 antibodies at 4°C overnight. Nonspecific IgG antibody precipitated the complex as a control group. To analyze the protein expression obtained by immunoprecipitation using anti-Bmal1 or S100A9 antibodies, we conducted western blot analysis. The input and IgG groups were regarded as positive and negative controls for the experiment, severally.

### Western Blot Analysis

Lysing liver tissues and RAW264.7 cells with RIPA buffer solution for extraction of protein by adopting centrifugal separation. After collection of supernatants, a BCA protein assay kit (Boster, China) calculated protein concentration. Protein lysates which were denatured by SDS underwent SDS-PAGE electrophoresis and were transferred to the 0.22 µm PVDF membrane (Roche, Penzber, Germany). The PVDF membranes were nonspecifically sealed with 5% milk and then washed with TBST (TBS contained 0.1% Tween-20) three times for 10 min each time. Following incubation with the special primary antibodies at 4°C overnight, then the PVDF membranes were flushed three times with TBST buffer. The membranes were then incubated with HRP-labeled goat antibodies against rabbit and mouse IgG (1:5,000, Beijing Zhongshan Biotechnology Co. Ltd., Beijing, China). After the preliminary treatment, the ECL-chemiluminescent kit (ECL, Advansta, United States) was used for detection. The densities of the protein immune response bands were analyzed with image J computer software. The primary antibodies were listed below: Bmal1, IL-10, IL-1β (Affinity Biosciences, Cincinnati, OH, United States), TNF-α, HK2, PFKP, S100A9 (Proteintech, Wuhan, China), ARG-1 (Wanleibio, Shenyang, China).

### Quantitative Real-Time PCR

TRIzol reagents were used to extract the total RNA according to the instructions. Reverse transcription of total RNA through cDNA synthesis was conducted by a kit. The reverse transcription reaction was performed for 15 min at 37°C and for 5 s at 85°C. The mRNA expression levels of Bmal1, TNF-α, IL-1β, IL-10, ARG-1, HK2, PFKP, and β-actin were detected by real-time quantitative PCR that was carried out by SYBR^®^ PrimeScript™ RT-PCR Kit (Takara, Kusatsu, Japan). The β-actin mRNA expression was considered an internal control. The primer sequences were shown as follows: Bmal1, 5′-AGT ACG TTT CTC GAC ACG CAA TAG-3′(forward) and 5′-TGT GGT AGA TAC GCC AAA ATA GCT-3′(reverse); TNF-α, 5′-CAC CAC CAT CAA GGA CTC AA-3′(forward) and 5′-AGG CAA CCT GAC CAC TCT CC-3′(reverse); IL-1β, 5′-CTT TGA AGT TGA CGG ACC C-3′(forward) and 5′-TGA GTG ATA CTG CCT GCC TG-3′(reverse); IL-10, 5′-GCC TTG CAG AAA AGA GAG CT-3′(forward) and 5′-AAA GAA AGT CTT CAC CTG GC-3′(reverse); ARG-1, 5′-CTC CAA GCC AAA GTC CTT AGA G-3′(forward) and 5′-AGG AGC TGT CAT TAG GGA CAT C-3′(reverse); HK2, 5′-TGA TCG CCT GCT TAT TCA CGG-3′(forward) and 5′-AAC CGC CTA GAA ATC TCC AGA-3′(reverse); PFKP, 5′-GCC GTG AAA CTC CGA GGA A-3′(forward) and 5′-GTT GCT CTT GAC AAT CTT CTC ATC AG-3′(reverse). The primers for β-actin were 5′-AGT GTG ACG TTG ACA TCC GT-3′(forward) and 5′-TGC TAG GAG CCA GAG CAG TA-3′(reverse). Each sample had a corresponding cycle threshold (Cq) value, and the 2^−ΔΔCt^ method was used to calculate the relative mRNA level.

### Mouse Bmal1 Plasmid Construction

The Bmal1 overexpressed plasmid (Bmal1-OE) was purchased from the GenePharma (shanghai, China). Abnormal expression of Bmal1 was achieved by transfection using Bmal1-OE, and empty vector was used as negative control. The constructed plasmids were transfected into RAW264.7 cells and mRNA expression of Bmal1 was verified by q RT-PCR.

### RNA Interference

Using Lipofectamine^®^ (2093076, Invitrogen, Carlsbad, CA) to interfere RNA as the instructions described. Small interfering RNA (siRNA) oligonucleotides against S100A9 were synthesized by GenePharma (Shanghai, China). Meanwhile, a negative scrambled siRNA (GenePharma, Shanghai, China) was used in parallel as a control. First, RAW264.7 cells were cultured in serum-free DMEM medium for 12 h and then reverse-transfected using the siRNA-S100A9 or the scrambled sequences of Opti-MEM (Gibco, United States). To replace normal culture medium after transfected 6 h later, then stimulated cells with LPS (1 µg/ml). The siRNA sequences were as follows: siRNA-S100A9 (mouse), 5′-CUG​AGC​AAG​AAG​GAA​UUC​ATT-3′ (sense) and 5′-UGA​AUU​CCU​UCU​UGC​UCA​GTT-3′ (antisense). All the above experiments were repeated three times.

### Statistical Analysis

Statistical Package for Social Sciences (SPSS Inc., Chicago, IL, United States, version 13.0) software was used for statistical analysis. The above results included data from at least three trials and were expressed as mean ± SD. One-way ANOVA was used to evaluate the results statistically. *p* < 0.05 was viewed as statistically significant.

## Results

### Establishment and Identification of ALD Model Induced by Alcohol in C57BL/6J mice

To observe whether the mice model was constructed successfully in EtOH-fed C57BL/6J mice, we performed histopathological analysis. In the first place, H&E staining was used to evaluate the extent of liver injury in EtOH-fed mice. The analysis showed that compared with the control diet (CD)-fed mice, the degree of liver injury was worse in the EtOH-fed mice. Fat vacuoles, intercellular distension, and cords disorder were appeared in liver tissues of EtOH-fed mice. The CD-fed mice, however, presented normal liver morphology with radiating cords and central veins (Supplementary Figure S1A). Corresponding to above results, the amount of lipid droplet deposition in the liver tissue of the EtOH-fed mice was significantly higher than that of the CD-fed group (Supplementary Figure S1B). To make sure the effect of alcohol intake on lipid balance, we assessed liver steatosis variation in ALD. As it turned out that the levels of TG and TCH in serum showed obviously ascending trend after alcohol intake (Supplementary Figure S1C). Moreover, the ratio of liver to body weight in EtOH-fed group was significantly higher than CD-fed group (Supplementary Figure S1D). What’s more, these lipid metabolic variations were closely related to the elevated expression of ALT and AST in serum (Supplementary Figure S1E). To sum up, ALD mouse model with mild to moderate inflammation and liver injury was successfully established after alcohol feeding.

### Down-Regulation of Bmal1 in EtOH-Fed Mice and Ethanol-Induced RAW264.7 Cells

**FIGURE 1:**
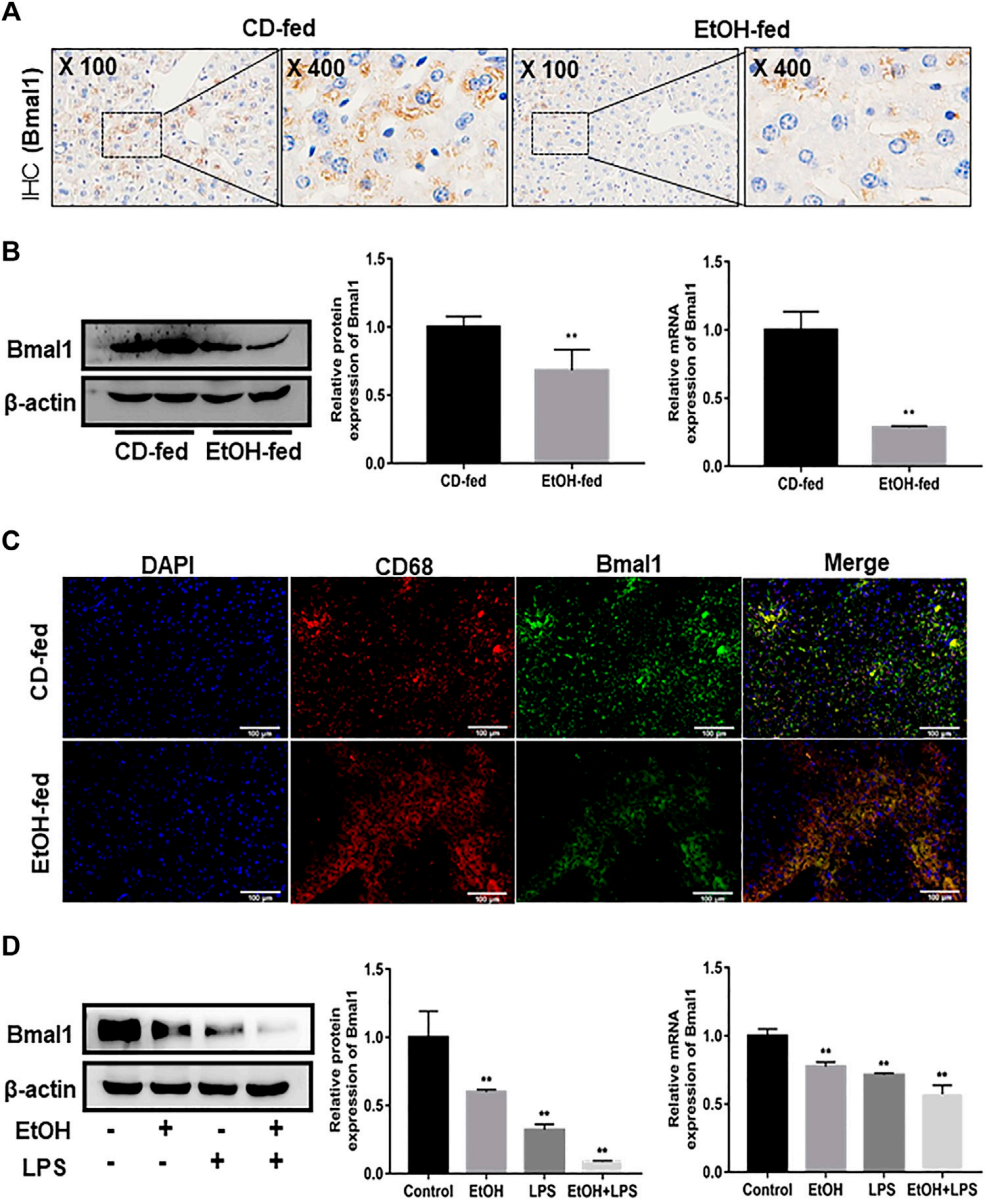
Effects of ethanol on Bmal1 expression in liver tissue and RAW264.7 cells. **(A)** The expression of Bmal1 in liver tissues was detected by IHC analysis. Each group presented a representative view. **(B)** The Bmal1 protein and mRNA levels in liver tissues were analyzed by western blot and q RT-PCR. **(C)** Immunofluorescence double-staining (IF) was used to analyze the representative co-localization of the immune reactivity of Bmal1 and macrophage CD68 in liver tissues. **(D)** The Bmal1 protein and mRNA levels in ethanol-induced RAW264.7 cells were analyzed by western blot and q RT-PCR. The results are shown as relative expression against control expression without treatment. Data shown are the mean ± SD from three independent experiments. *p < 0.05, **p < 0.01 vs. CD-fed group or control group.

It was known that Bmal1 was involved in the regulation of inflammatory responses. Hence, to verify the influence of ethanol on the expression pattern of Bmal1, IHC analysis, western blot and q RT-PCR were performed, respectively. Bmal1 was highly expressed in liver tissues of CD-fed mice, while a nonnegligible decrease occurred in EtOH-fed group (Figures 1A,B). In addition, immunofluorescence double-staining analysis was conducted to manifest the presence of typical Bmal1 co-localization with macrophage CD68 immunoreactivity in liver tissues (Figure 1C). To further demonstrate that ethanol can inhibit the expression of Bmal1, macrophages were stimulated with ethanol of 25 mM for 24 h *in vitro*. Ethanol markedly diminished the protein and mRNA expression of Bmal1 in RAW264.7 cells as analyzed by western blot and q RT-PCR. Significantly, this effect was further enhanced by the addition of 1 µg/ml LPS stimulation in the presence of ethanol (Figure 1D). All of the above results indicated that ethanol stimulation inhibits the expression of Bmal1. When LPS was added to induce RAW264.7 cells *in vitro*, the suppression of Bmal1 expression was more pronounced.

### Characteristics of Macrophage Phenotypes in EtOH-Fed Mice and Ethanol-Induced RAW264.7 cells

**FIGURE 2:**
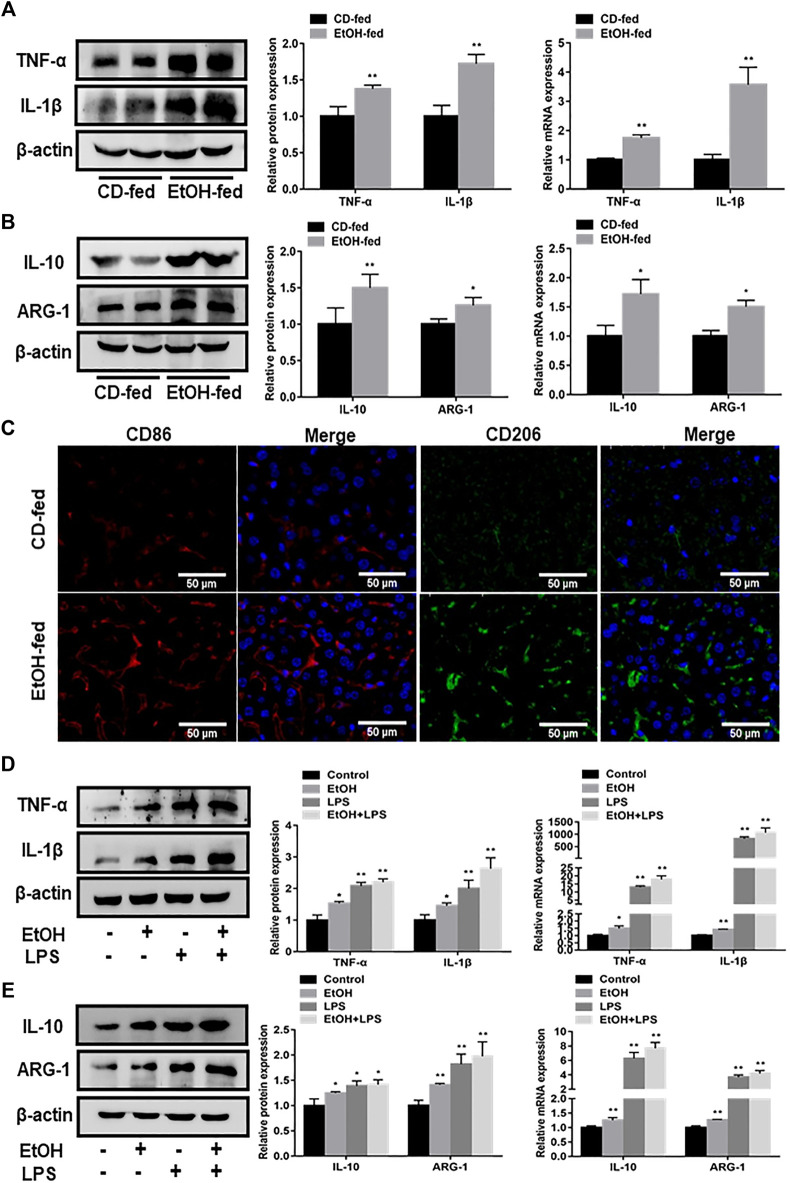
Phenotypic characteristics of macrophages in EtOH-fed mice liver tissue and ethanol-stimulated RAW264.7 cells. **(A)** Effects of ethanol on protein and mRNA levels of M1 macrophage markers (TNF-α, IL-1β) in liver tissue. **(B)** The protein and mRNA levels of M2 macrophage markers (IL-10, ARG-1) in liver tissue. **(C)** Immunofluorescence staining results of M1, M2 macrophage markers (CD86, CD206) in liver tissue of EtOH-fed mice. **(D)** The protein and mRNA levels of M1 macrophage (TNF-α, IL-1β) in ethanol-induced RAW264.7 cells. **(E)** The protein and mRNA levels of M2 macrophage markers (IL-10, ARG-1) in ethanol-induced RAW264.7 cells. The results are shown as relative expression against control expression without treatment. Data shown are the mean ± SD from three independent experiments. *p < 0.05, **p < 0.01 vs. CD-fed group or control group.

In order to determine the features of macrophage phenotypes in EtOH-fed mice and ethanol-stimulated RAW264.7 cells, we detected the expression of macrophage surface markers by using western blot and q RT-PCR analysis. The protein and mRNA results presented that liver tissues in EtOH-fed group showed a distinct augment of M1 macrophage markers (TNF-α, IL-1β) levels compared with CD-fed group (Figure 2A). Moreover, the expression of M2 macrophage markers (IL-10, ARG-1) were also revealed an upward trend (Figure 2B). Notably, the liver tissue immunofluorescence staining results also demonstrated that both M1 and M2 macrophage markers (CD86, CD206) increased in the alcohol-fed mice (Figure 2C). We further investigated alteration of macrophage surface markers *in vitro* ethanol-induced RAW264.7 cells. Results indicated that the protein and mRNA expression of TNF-α, IL-1β were upregulated after RAW264.7 cells treated by ethanol. Moreover, compared with the ethanol group, the increase of ethanol plus LPS group was more obvious (Figure 2D). The M2 macrophage markers (IL-10, ARG-1) also showed the same change trend (Figure 2E). Importantly, flow cytometry showed that M1, M2 macrophage markers (CD86, CD206) also increased *in vitro* (Supplementary Figure S2A). Therefore, we supposed that ethanol induced the polarization of M1/M2 macrophages *in vivo* and vitro. In addition, the increase of M1 macrophages was more obvious than that of M2-type.

### Bmal1 Regulates the M1/M2 Phenotype Transformation of Macrophages *in vitro*

**FIGURE 3:**
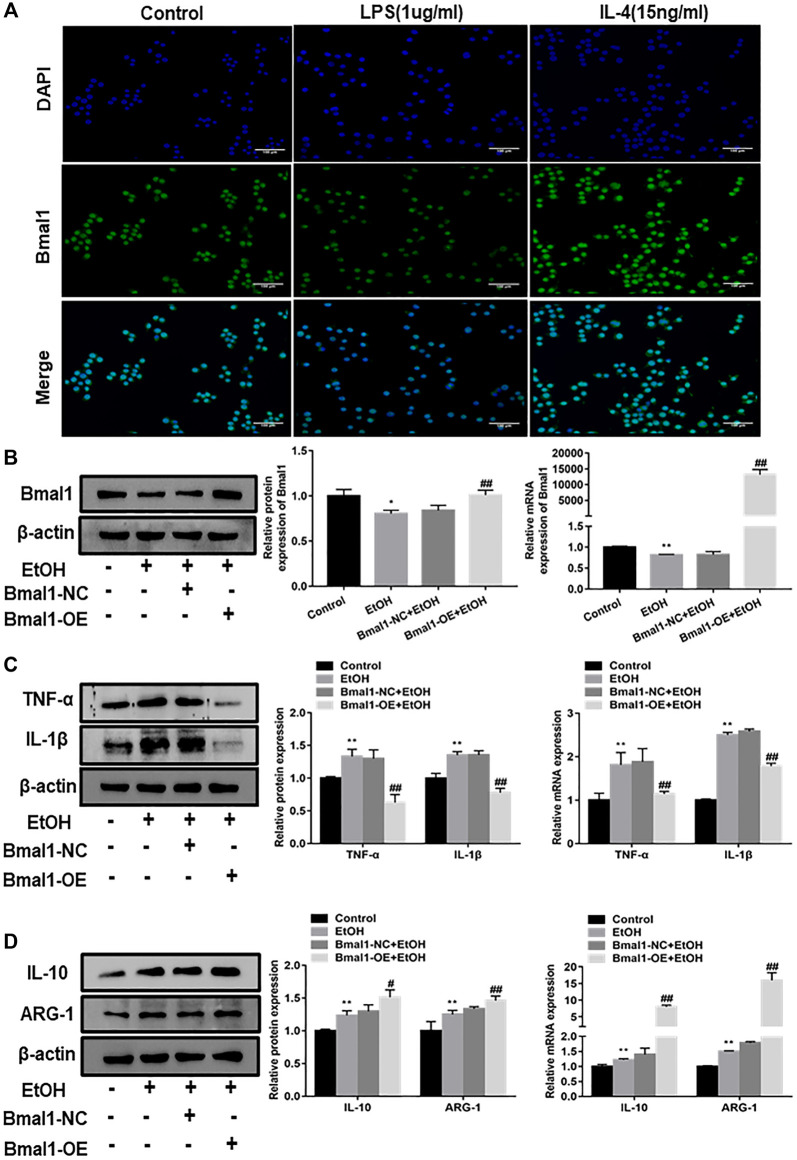
Effect of Bmal1 overexpression on macrophage polarization. **(A)** The expression of Bmal1 in RAW264.7 macrophages polarization was analyzed by immunofluorescence (IF) assay. **(B)** Bmal1 successful over-expression was confirmed by western blot and q RT-PCR in ethanol-induced RAW264.7 cells. The results are shown as relative expression against control expression without treatment. **(C)** The protein and mRNA levels of M1 macrophages biomarkers (TNF-α, IL-1β) were analyzed by western blot and q RT-PCR. **(D)** The protein and mRNA levels of M2 macrophages biomarkers (IL-10, ARG-1) were analyzed by western blot and q RT-PCR. The results are shown as relative expression against control expression without treatment. Data shown are the mean ± SD from three independent experiments. *p < 0.05, **p < 0.01 vs. control group. ^#^ *p* < 0.05, ^##^ *p* < 0.01 vs. Bmal1-NC + EtOH group.

In order to further analyze whether Bmal1 participated in the progress of macrophage polarization, RAW264.7 cells were polarized into M1 macrophage phenotypes by stimulated with LPS or treated with IL-4 to induce M2-type macrophages. Immunofluorescence analysis indicated that the fluorescence intensity of Bmal1 in RAW264.7 cells treated with IL-4 was significantly higher than control group, however, LPS stimulation significantly reduced the fluorescence intensity (Figure 3A). It can be seen from this that LPS stimulation led to the downregulation of Bmal1 in RAW264.7 cells. In contrast, Bmal1 was elevated in RAW264.7 cells treated with IL-4. In summary, these results suggested that Bmal1 may be involved in macrophage phenotypic alteration.

To explore whether Bmal1 could affect macrophage polarization, we overexpressed Bmal1 by transfected Bmal1 plasmids into ethanol stimulated RAW264.7 cells. After transfection of the Bmal1 plasmid, the protein and mRNA levels of Bmal1 were significantly increased, which means that the Bmal1 overexpression model was successfully constructed (Figure 3B). It's probably worth noting that the protein and mRNA expression of M1 macrophage markers including TNF-α, IL-1β decreased after the overexpression of Bmal1 compared with Bmal1-NC group (Figure 3C). In contrast, Bmal1 overexpression further up-regulated the protein expression of M2 macrophage surface markers such as IL-10, ARG-1. In addition, mRNA levels of IL-10 and ARG-1 increased synchronously with protein levels (Figure 3D). From the above results, we considered that Bmal1 affects the process of macrophage polarization.

### Bmal1 Regulates M1 Polarization of Macrophages by Inhibiting Glycolysis

**FIGURE 4:**
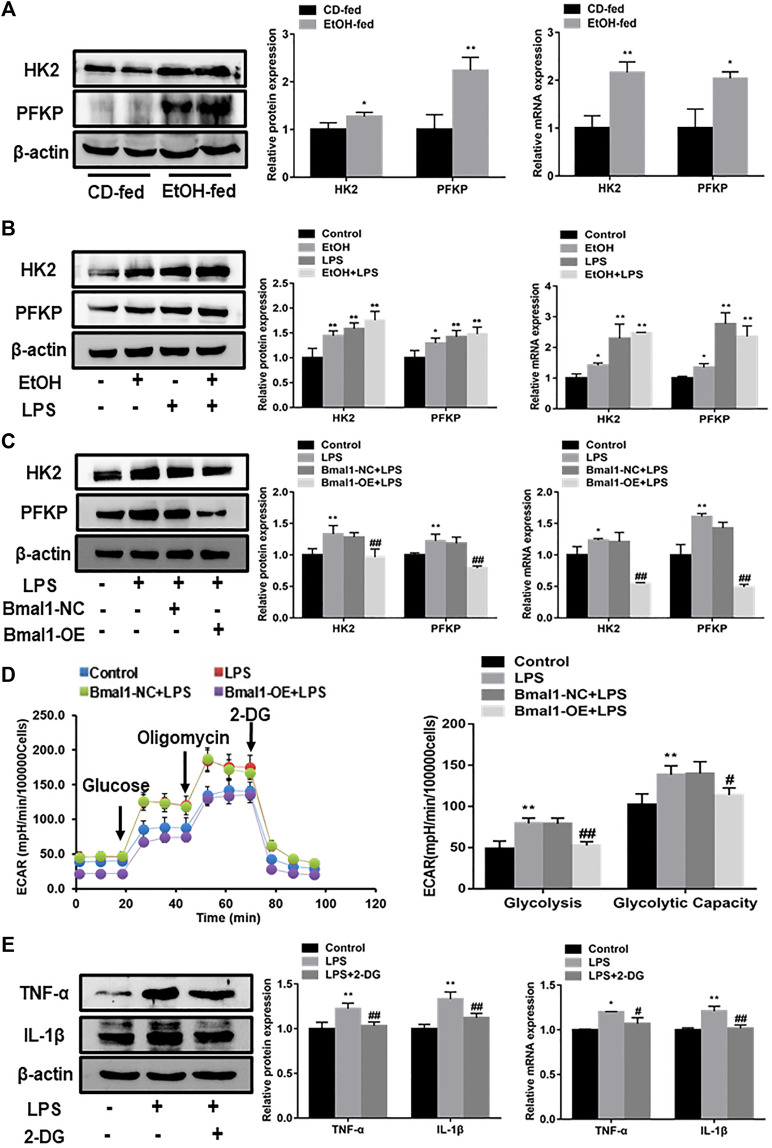
Interaction of Bmal1 with glycolysis in mouse macrophages. **(A)** The protein and mRNA expression of glycolytic key enzyme (HK2, PFKP) were observed in liver tissue by western blot and q RT-PCR. **(B)** The protein and mRNA expression of glycolytic key enzyme (HK2, PFKP) were observed in ethanol-induced RAW264.7 cells by western blot and q RT-PCR. **(C)** Effect of Bmal1 overexpression on glycolytic key enzymes levels in LPS-stimulated RAW264.7 cells determined by western blot and q RT-PCR. **(D)** The extracellular acidification rate (ECAR) of Bmal1-OE transfected RAW264.7 cells was detected by Seahorse XF Analyzer, and normalized into cell number. **(E)** Effect of 2-DG on M1 macrophage biomarkers expression in LPS-stimulated RAW264.7 cells. The expression of M1 macrophage biomarkers (TNF-α, IL-1β) was determined by western blot and q RT-PCR. Data shown are the mean ± SD from three independent experiments. ^*^ *p* < 0.05, ^**^ *p* < 0.01 vs. CD-fed group or control group. ^#^ *p* < 0.05, ^##^ *p* < 0.01 vs. Bmal1-NC + LPS group.

To get better acquainted with the mechanism by which Bmal1 regulated macrophage polarization, we looked for a signaling pathway which was involved in the process of macrophage polarization caused by Bmal1. It was well known that the glycolytic pathway participated in the correlated process of macrophage polarization. In accordance with expectation, glycolytic pathway was activated in the liver tissue of EtOH-fed mice and ethanol stimulated RAW264.7 cells, estimated by an increase in protein and mRNA expression of key glycolytic enzymes such as HK2, PFKP (Figures 4A,B). Furthermore, lactate levels were significantly higher in the serum of EtOH-fed mice and ethanol induced cell supernatant (Supplementary Figures S3A,B). In particular, HK2 and PFKP protein levels were dramatically increased in LPS induced M1 macrophages, while IL-4 induced M2 macrophages showed no clear differences (Supplementary Figures S3C,D). These consequences indicated that the glycolytic pathway was abnormally activated when RAW264.7 cells was treated with ethanol and mice were fed with ethanol. In addition, the reduced trend was observed in the expression of key enzymes (HK2, PFKP) protein and mRNA when overexpressed Bmal1 in LPS induced macrophage (Figure 4C). Moreover, the level of lactic acid released into cellular supernatant also declined (Supplementary Figure S3E). Most notably, ECAR were inhibited in the Bmal1-OE group compared with the Bmal1-NC group *in vitro* LPS-induced macrophages. Among them, the basal glycolysis and glycolytic capacity were declined sharply (Figure 4D).

To further assess whether glycolytic pathway was conducive to the polarization of M1 macrophages, a glycolytic chemical inhibitor 2-deoxy D glucose (2-DG), was used to inhibit the glycolytic pathway in M1 macrophages. The results showed that the key glycolytic enzymes (HK2, PFKP) protein levels in LPS-induced macrophages were evidently restrained after the presence of 2-DG (Supplementary Figure S3F). It can be seen from the results that 2-DG obviously suppresses the expression of M1 macrophage markers such as TNF-α, IL-1β (Figure 4E). Notably, the inhibition of Bmal1 on M1 macrophage markers protein was reversed by the use of oligomycin for activating glycolysis after overexpression of Bmal1 (Supplementary Figure S3G). Therefore, we concluded that Bmal1 negatively regulates the expression of M1-type macrophage markers by inhibiting glycolysis.

### The Interaction Between Bmal1 and S100A9 Result in the Obstruction of Glycolytic Pathway, Modulating M1 Macrophage Polarization

**FIGURE 5:**
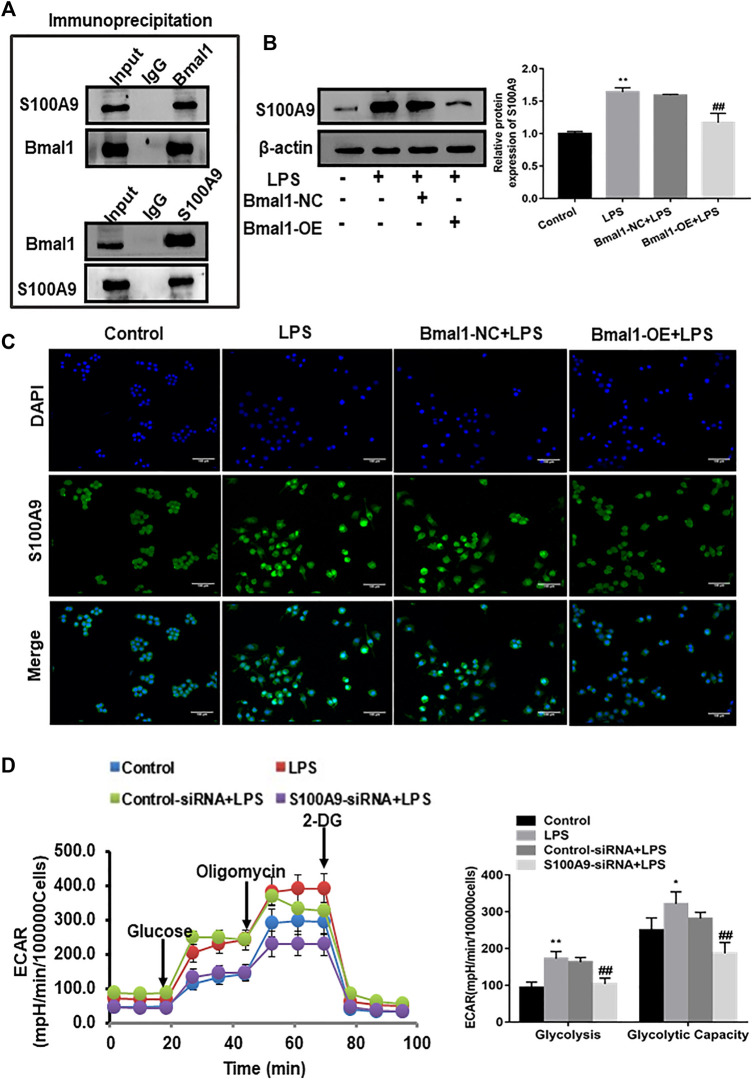
The role of S100A9 in the regulation of glycolytic pathway by Bmal1. **(A)** Co-IP of Bmal1 and S100A9 in M1 macrophages using the anti-Bmal1 and anti-S100A9 antibody. **(B)** The protein level of S100A9 in Bmal1-OE group was analyzed by western blot. **(C)** The expression of S100A9 was analyzed by immunofluorescence (IF) assay in LPS-induced RAW264.7 cells after Bmal1 overexpression. **(D)** The extracellular acidification rate (ECAR) of S100A9 siRNA transfected RAW264.7 cells was detected by Seahorse XF Analyzer, and normalized into cell number. Data shown are the mean ± SD from three independent experiments. *p < 0.05, **p < 0.01 vs. control group. ^#^ *p* < 0.05, ^##^ *p* < 0.01 vs. Bmal1-NC + LPS or control siRNA + LPS group.

Based on the above exploration, we continued to investigate which proteins interacted with Bmal1 to block glycolysis and suppress M1 polarization. Through Co-IP experiment, Bmal1 directly bound with S100A9 protein to have effect on M1 macrophages (Figure 5A). It had been reported that S100A9 which was highly expressed under inflammatory conditions could recruit macrophages. Just as we expected, the expression of S100A9 up-regulated, confirmed by increased protein levels, which was observed in the liver of EtOH-fed mice (Supplementary Figure S4A). The expression of S100A9 protein in RAW264.7 cells was significantly up-regulated after ethanol stimulation, and the up-regulated extent was increased after the addition of LPS (Supplementary Figure S4B).

According to previous reports, S100A9 alone could enhance glycolysis of neutrophils. Next, in order to prove that Bmal1 affected the glycolysis pathway in M1 macrophage through S100A9, western blot analysis revealed that overexpression of Bmal1 significantly cut down the expression of S100A9 in LPS-induced RAW264.7 cells (Figure 5B). Moreover, S100A9 fluorescence was also weaker in the Bmal1-OE group than in the Bmal1-NC group of LPS-induced macrophage (Figure 5C).

In fact, to further verify whether S100A9 was responsible for glycolysis in M1 macrophages, siRNA was selected to block S100A9 expression in RAW264.7 cells stimulated by LPS. When S100A9 was silenced, ECAR real-time detection results demonstrated that both basal and glycolytic capacities were reduced (Figure 5D). At the same time, the protein expression of key glycolytic enzymes such as HK2 and PFKP also showed a downward trend (Supplementary Figure S4C). Importantly, the decreased expression of key glycolytic enzymes associated with overexpression of Bmal1 were significantly reversed after overexpression of S100A9. The same trend was seen in the lactic acid secreted by cells (Supplementary Figure S4D). As expected, S100A9 silencing resulted in the inhibition of M1 macrophage markers expression (Supplementary Figure S4E). In short, these data reflected the interaction between Bmal1 and S100A9 that modulated glycolysis to affect M1 macrophage polarization.

### Hepatic Specific Overexpression of Bmal1 Alters Characteristics of the Macrophage Phenotypes *In Vivo*

**FIGURE 6:**
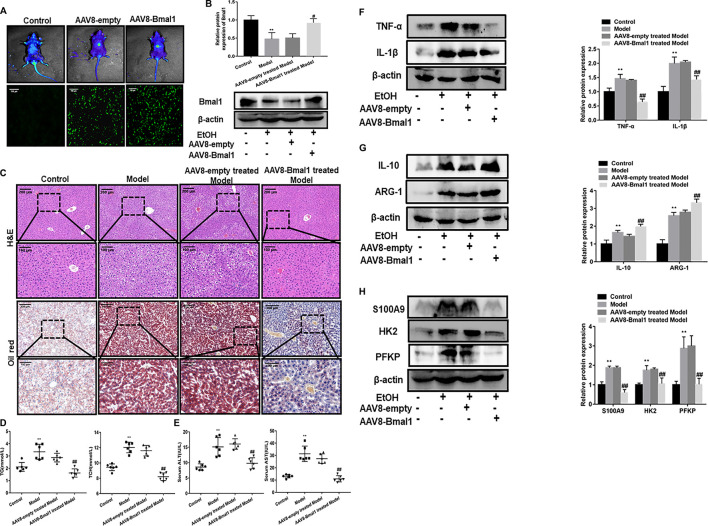
Liver-specific Bmal1 overexpression alleviates ethanol induced liver injury in mice. **(A)** Representative efficient transduction of rAAV8–Bmal1-eGFP in liver tissues by fluorescent microscopy and living imaging. **(B)** Western blot analysis of Bmal1 protein expression in liver tissue after AAV8 injection. **(C)** Representative hematoxylin and eosin (H&E) staining and Oil red O staining of liver tissues. Representative views from each group are presented. **(D)** Serum triglyceride and total cholesterol levels in mice. **(E)** Serum ALT and AST levels in mice. **(F)** The protein expression of M1 macrophage markers after AAV8 injection. **(G)** The protein expression of M2 macrophage markers after AAV8 injection. **(H)** The protein expression of S100A9 and glycolytic key enzymes after AAV8 injection. The values represent means ± SD. (n = 6 in each group) *p < 0.05, **p < 0.01 vs. control group. ^#^ *p* < 0.05, ^##^ *p* < 0.01 vs. AAV8-empty treated Model group.

Recently, the evolution of recombinant AAV (rAAV) vector which root in alternative serotype as an effective method to transmit genes targeting tissues, has attracted much attention. More importantly, with the development of this technology, recombinant vector can be effectively transduced into the liver. In EtOH-fed mice, we also needed to determine if the adenovirus was injected into the mouse liver and expressed effectively. As indicated by eGFP expression that was detected by fluorescence microscopy, rAAV8 was effectively transferred to the liver after 21 days of injection. Moreover, *in vivo* fluorescence imaging results also confirmed this (Figure 6A). Importantly, Bmal1 protein levels were significantly elevated *in vivo* after AAV8 injection (Figure 6B). What’s more, histopathological examination of H&E staining and Oil Red O staining showed that liver injury and lipid deposition were alleviated to some extent after rAAV8-Bmal1 injection (Figure 6C). Similarly, serum TG and TCH levels were evidently down-regulated in EtOH-fed mice treated with rAAV8-Bmal1 (Figure 6D). Furthermore, serum ALT and AST increased in mice treated with empty rAAV8, while decreased in mice treated with rAAV8-Bmal1 (Figure 6E).

We further investigated the effect of Bmal1 overexpression on macrophage phenotypes to verify the role of Bmal1 in EtOH-fed mice. The results turned out that the levels of M1 macrophage markers TNF-α, IL-1β in EtOH-fed mice with overexpression of Bmal1 was visibly declined (Figure 6F). In contrast, M2 macrophage markers (IL-10, ARG-1) expression increased notably (Figure 6G). In addition, abnormal overexpression of Bmal1 *in vitro* also significantly antagonized the expression of key glycolytic enzymes and S100A9 (Figure 6H). Importantly, Bmal1 and macrophage markers fluorescence in liver tissue of mice injected with AAV8 indicated upregulation of Bmal1 and alteration of macrophage markers *in vivo* (Supplementary Figures S5A,B). These results indicated that Bmal1 inhibits glycolysis by interacting with S100A9, which may provide a possible explanation for ethanol-induced liver disease and M1 macrophage polarization *in vitro*.

## Discussion

Upon ALD, it has been one of the most common liver diseases in the world, ranging from AFL, alcoholic hepatitis, fibrosis and cirrhosis (Gao and Bataller, 2011; Osna et al., 2017). Many studies have depicted ALD is characterized by extensive steatosis accompanied with chronic inflammation (Park, 2014; Szabo, 2015; Kawaratani et al., 2017). Moreover, activated macrophages can further trigger the inflammatory response process by releasing cytokines, chemokines (Watanabe et al., 2019). Therefore, the regulation of inflammatory response by affecting macrophage polarization may be an important measure in the treatment of ALD. Bmal1, a core gene in the body clock, regulates the liver’s biological rhythms. Importantly, it has been pointed out in the literature that the clock gene Bmal1 has crucial effect on macrophages in a variety of diseases, such as inflammatory and metabolic diseases (Oishi et al., 2017; Alexander et al., 2020). Furthermore, inflammatory stimulant LPS can also suppress Bmal1 expression in the innate immune response of macrophages (Curtis et al., 2015). Therefore, we hypothesized that Bmal1 might influence the phenotypic changes of hepatic macrophages. However, the association between Bmal1 and macrophage polarization remains unclear.

Generally, our results suggested that Bmal1 expression was down-regulated in liver tissue of EtOH-fed mice and ethanol treated RAW264.7 cells. Previous studies have indicated that the imbalance of M1 and M2 macrophage polarization in the liver drives an inflammatory response to resist damage of organism (Bashir et al., 2016; Shapouri-Moghaddam et al., 2018). Unsurprisingly, there was a significant increase in macrophage markers expression. In addition, we further investigated that the effect of Bmal1 on liver injury induced by ethanol *in vivo* by injecting AAV8-Bmal1 vector, suggesting that while Bmal1 was overexpressed, the degree of lipid accumulation and liver damage were alleviated compared to mice injected with AAV8-empty vector. Importantly, the expression of M1 macrophage markers (TNF-α, IL-1β) was inhibited, but M2 markers (IL-10, ARG-1) were increased. Similarly, the over-expression of Bmal1 resulted in the phenotypes of macrophages have appeared the same trend *in vitro*. Consistent with the conjecture, Bmal1 has effect on the polarization of macrophages. Therefore, we concluded that Bmal1 may alleviates the degree of liver injury by regulating macrophage polarization in ALD.

Accumulating evidences have discovered that the transformation of energy metabolism seems to be closely related to macrophage polarization (Biswas and Mantovani, 2012). Among them, glycolysis is a key pathway (Zhu et al., 2015). Blocking the glycolytic pathway can prevent the release and expression of proinflammatory cytokines, thus affecting the differentiation of M1 macrophages (Li et al., 2018). In contrast, IL-4 induced M2 macrophages preferentially increase the oxidative phosphorylation rate *in vivo* (Wang et al., 2019). According to our results, the expression of glycolytic key enzymes and lactate was upregulated overall in both EtOH-fed mice and ethanol-stimulated macrophages. Consistent with the literature, data showed that glycolytic enzyme level was significantly up-regulated in M1 macrophages, while no significant variation in M2 macrophages. Therefore, we further confirmed whether the glycolytic pathway could impact M1 macrophage polarization. The use of glycolysis inhibitor 2-DG led to a significant reduction in the expression of M1 macrophage markers in LPS-stimulated RAW264.7 cells. Consequently, it can be seen that M1 macrophage polarization is positively modulated by glycolytic pathway.

Previous study shows that calcium binding protein S100A9, an inflammatory mediator, is involved in the progression of a variety of inflammatory diseases (Shabani et al., 2018; Wang et al., 2018). In addition, it promotes macrophage migration and mediates the inflammatory signal cascade reaction by binding with TLR4 and RAGE on the adjacent cell surface. In our study, a distinct augment of S100A9 protein was observed in the liver tissues of EtOH-fed mice as well as LPS-induced RAW264.7. Moreover, mass spectrometry screening showed that S100A9 could act as an intermediate protein for Bmal1 to interact with glycolysis. Reports have pointed out that S100A9 independently induce the increase of ECAR, indicating that it can enhance the rate of glycolysis in neutrophils (Rousseau et al., 2017). The ECAR values and protein expression of glycolytic key enzymes were found to be limited after silencing S100A9. Furthermore, S100A9 silencing also resulted in inhibition of M1 macrophage markers. Based on the experimental data discussed above, we summarize that S100A9 may promote M1 polarization by up-regulating glycolytic pathway.

In our current study, Co-IP results demonstrated that Bmal1 can bind with S100A9 to adjust glycolytic pathways. When Bmal1 is overexpressed, the protein expression and fluorescence intensity of S100A9 are significantly inhibited in LPS-stimulated RAW264.7 cells. After overexpression of Bmal1, EACR and protein expression of key enzymes showed that glycolysis was also obviously down-regulated. Furthermore, the downregulation of glycolysis induced by Bmal1 overexpression was reversed by upregulation of S100A9. It is worth noting that when exogenous Bmal1 is overexpressed, the macrophage phenotype changes correspondingly, mainly manifested as the decrease of M1 macrophage markers and the increase of M2 markers. In order to further confirm that Bmal1 regulates macrophage polarization through glycolysis, we added glycolytic agonist oligomycin after overexpression of Bmal1 to analyze the phenotypic changes of macrophages. The results showed that Bmal1 caused M1 macrophage inhibition, which was reversed after glycolysis was activated. In summary, this phenomenon directly verified that the interaction between Bmal1 and S100A9 indeed change the polarization state of macrophages through glycolysis.

**FIGURE 7:**
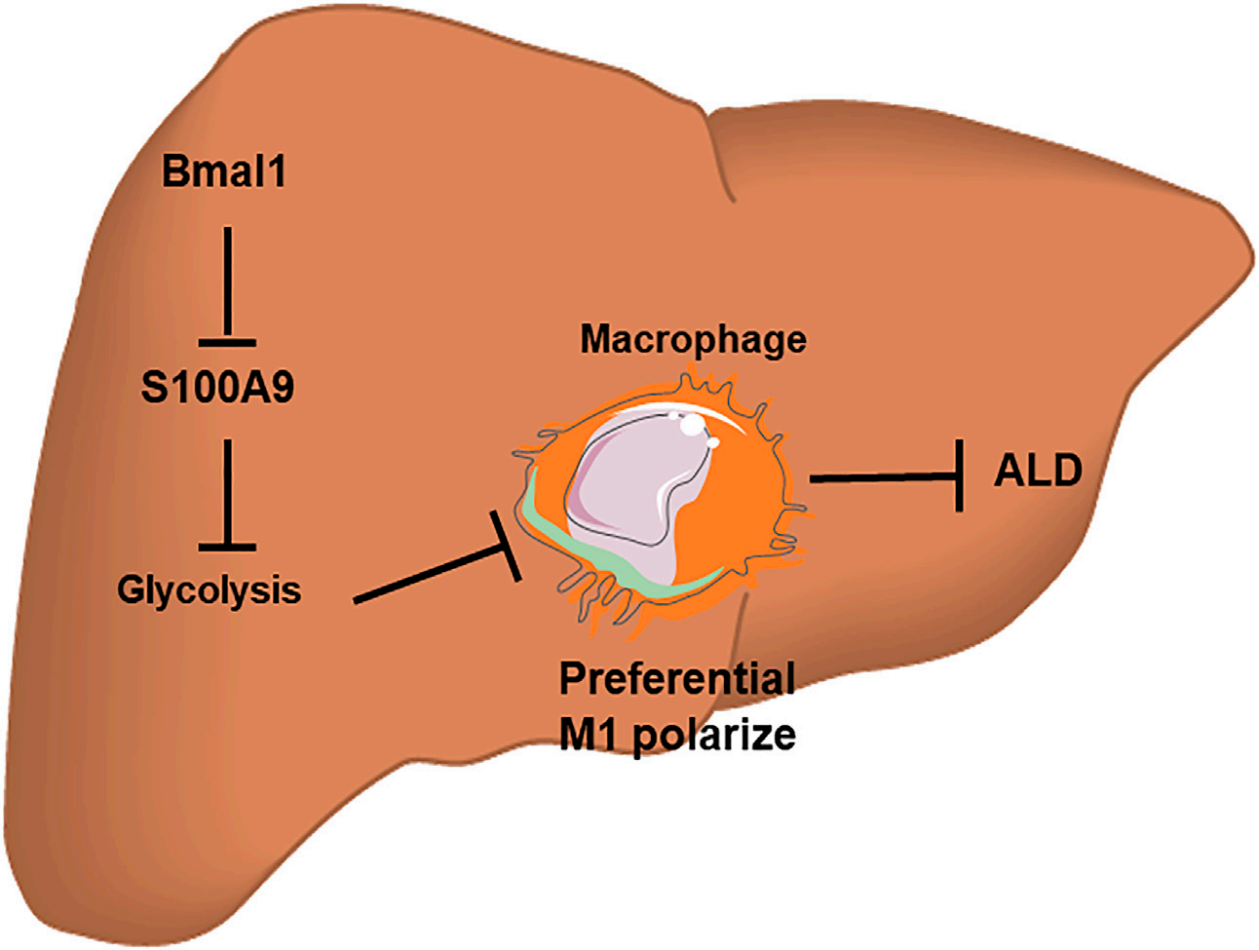
A schematic diagram of the molecular mechanisms by which Bmal1 acts with glycolytic pathway to regulate macrophage polarization in a chronic binge alcohol-induced ALD mouse model. The down-regulation of Bmal1 expression after EtOH treatment can induce the transformation of macrophages into M1-type by interacting with glycolytic pathway. However, the interaction between Bmal1 and glycolysis is linked by S100A9 protein. In brief, this crosstalk mechanism offers a possible interpretation for the inflammatory response causing by liver macrophage polarization induced by ethanol *in vitro*.

All in all, Bmal1 regulates macrophage polarization by targeting glycolysis, thereby improving the inflammatory response in ALD (Figure 7). Consequently, Bmal1 is expected to be an effective target for the prevention and improvement of ALD. Overexpression of Bmal1 may be a novel therapeutic strategy for ALD. This series of researches provides new ideas for evaluating disease progression and exploring targeted drugs.

## Data Availability

The raw data supporting the conclusions of this article will be made available by the authors, without undue reservation.
