# Supplementary figures and images for: Bmal1 Regulates Macrophage Polarize Through Glycolytic Pathway in Alcoholic Liver Disease
Source: Front Pharmacol. 2021 Mar 10;12:640521. doi: 10.3389/fphar.2021.640521 (PMC8006279; doi:10.3389/fphar.2021.640521)

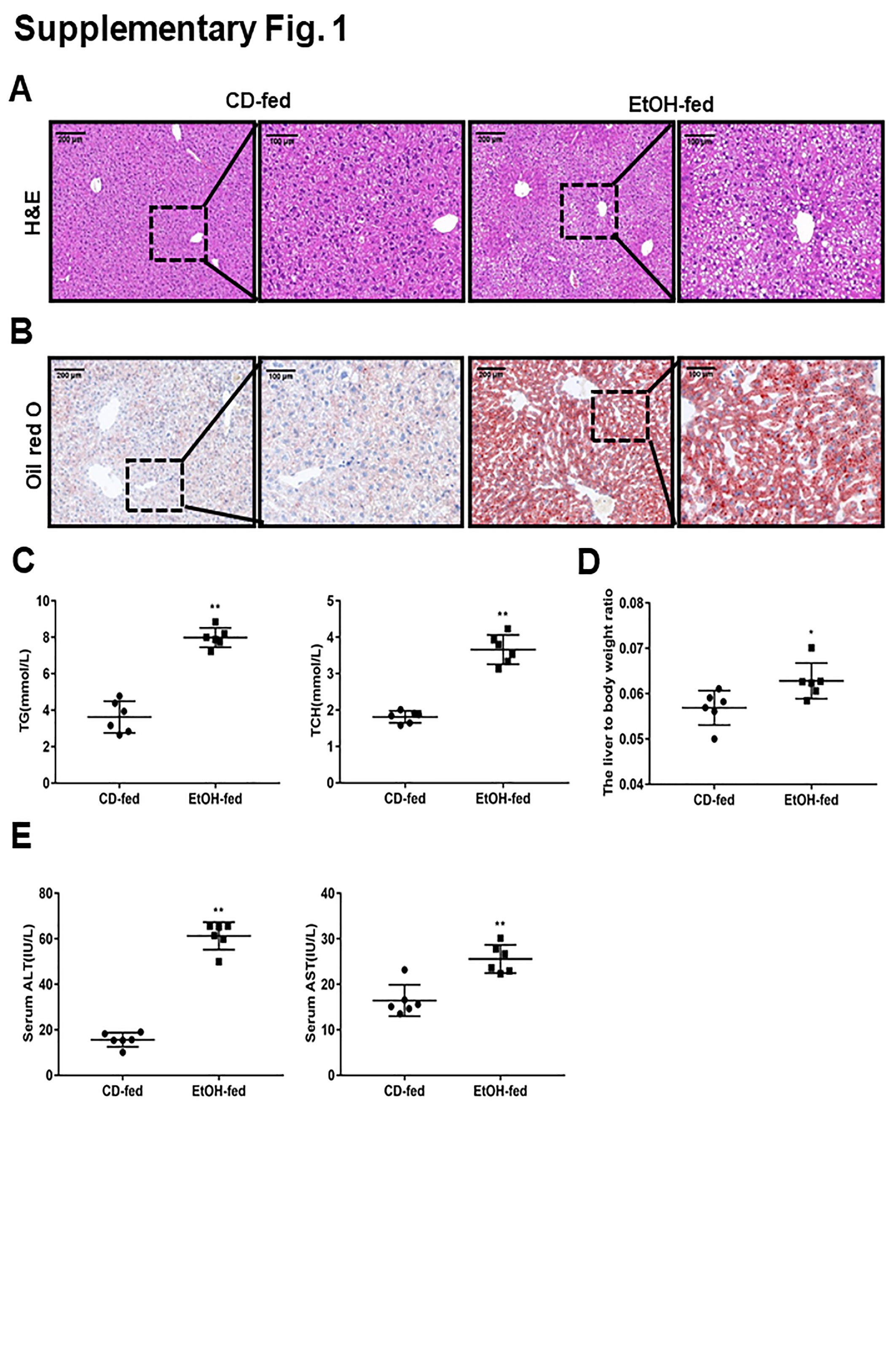

Supplement: Supplementary file 1 [file image1.tif]

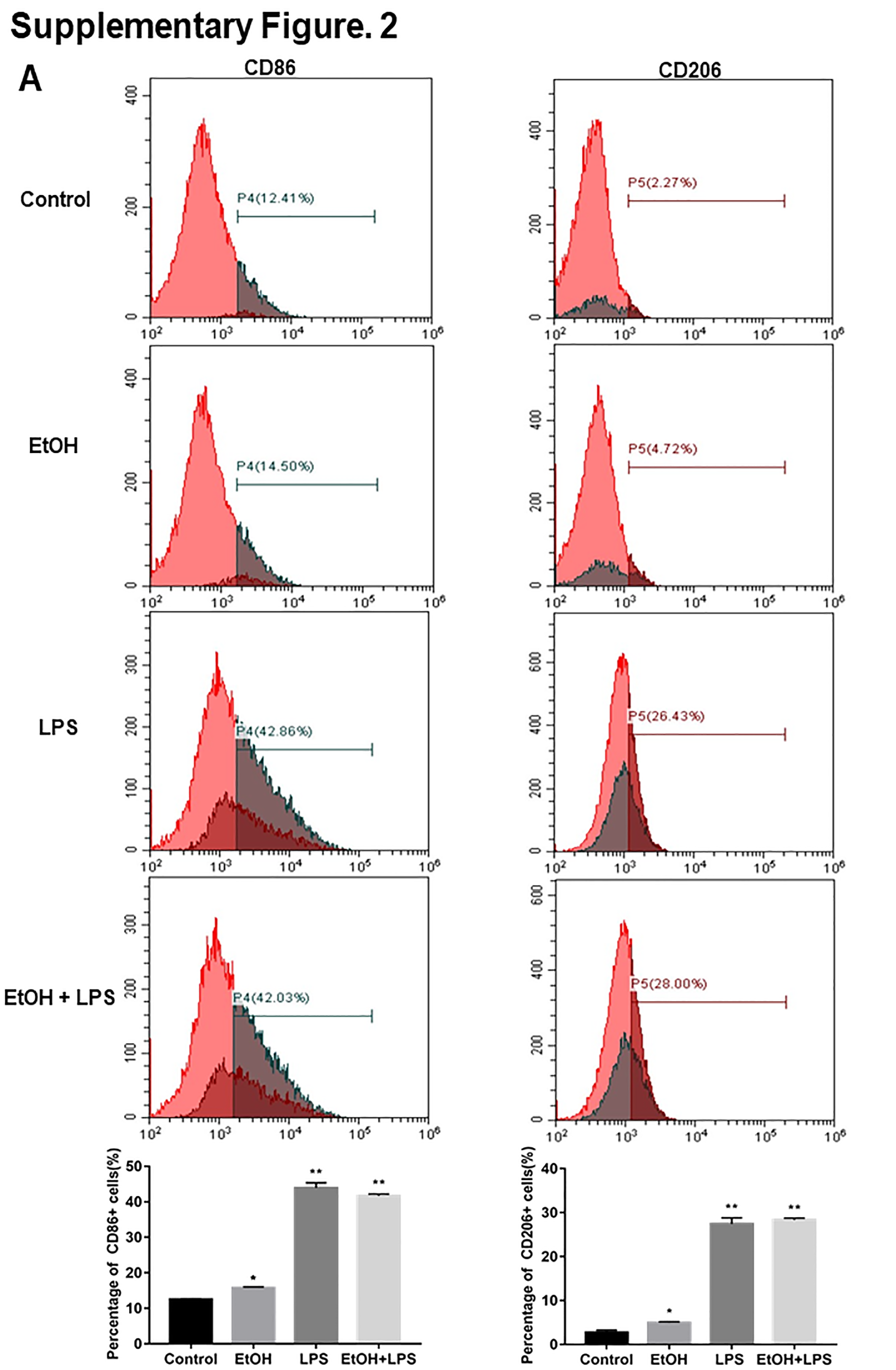

Supplement: Supplementary file 2 [file image2.tif]

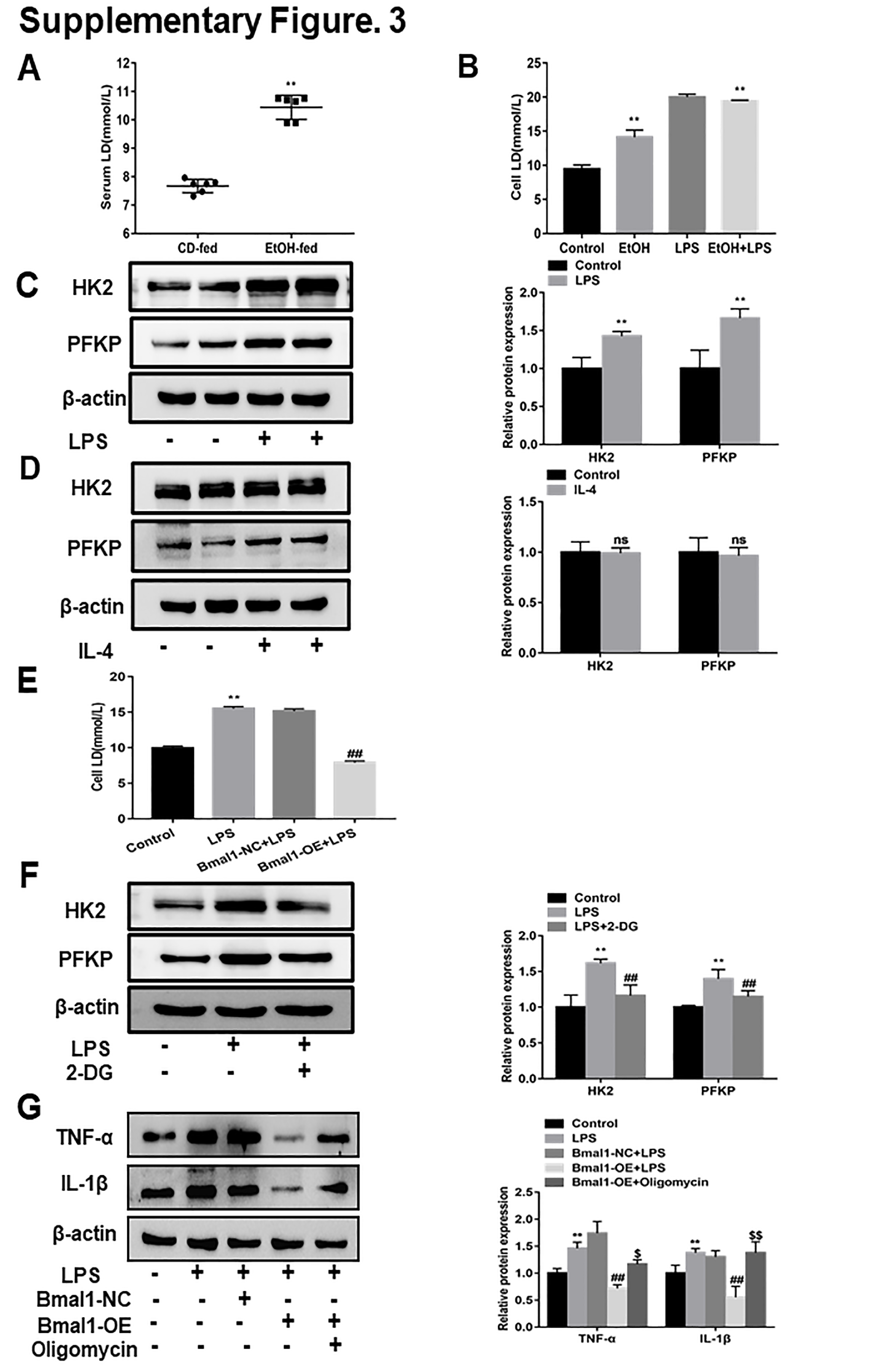

Supplement: Supplementary file 3 [file image3.tif]

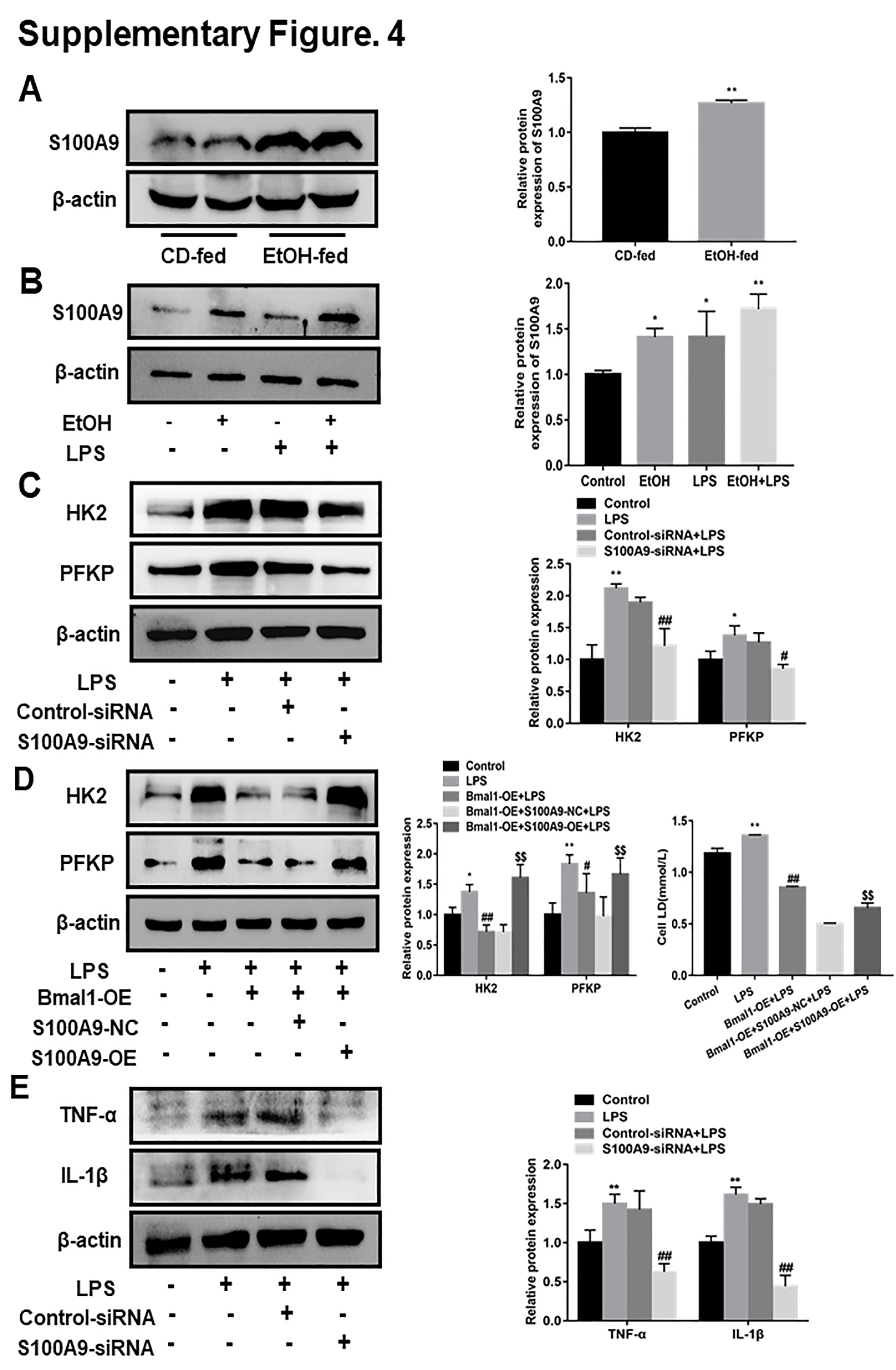

Supplement: Supplementary file 4 [file image4.tif]

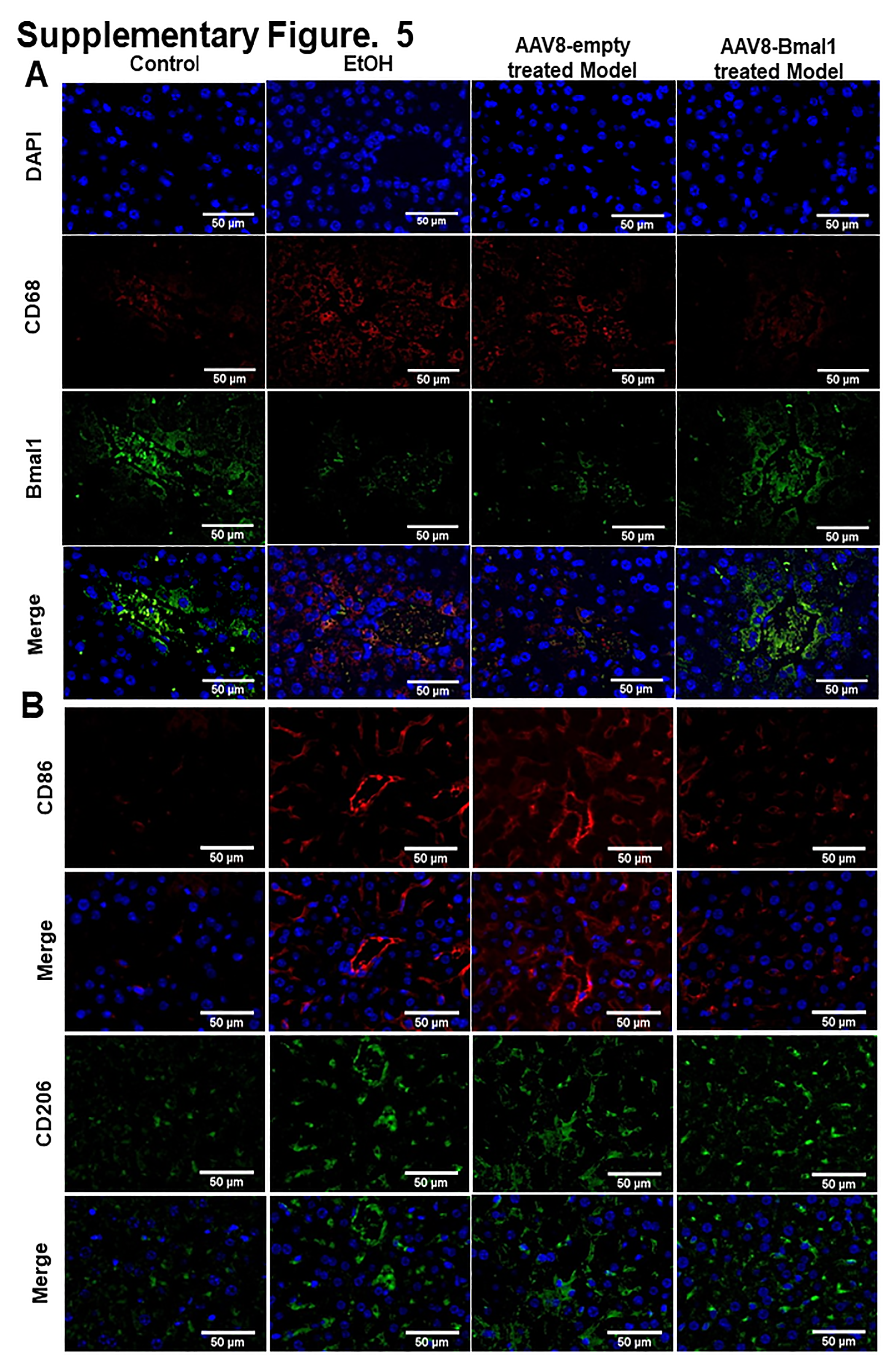

Supplement: Supplementary file 5 [file image5.tif]
